# In Silico Screening and Molecular Dynamics Simulation Studies in the Identification of Natural Compound Inhibitors Targeting the Human Norovirus RdRp Protein to Fight Gastroenteritis

**DOI:** 10.3390/ijms24055003

**Published:** 2023-03-05

**Authors:** Rami J. Obaid, Alaa Shafie, M. Shaheer Malik, Munirah M. Al-Rooqi, Ziad Moussa, Osama Abdulaziz, Abdulelah Aljuaid, Mamdouh Allahyani, Mazen Almehmadi, Farah Anjum, Saleh A. Ahmed

**Affiliations:** 1 Department of Chemistry, Faculty of Applied Sciences, Umm Al-Qura University, Makkah 21955, Saudi Arabia; 2 Department of Clinical Laboratory Sciences, College of Applied Medical Sciences, Taif University, P.O. Box 11099, Taif 21944, Saudi Arabia; 3 Department of Chemistry, College of Science, United Arab Emirates University, Al Ain P.O. Box 15551, United Arab Emirates; 4 Department of Chemistry, Faculty of Science, Assiut University, Assiut 71516, Egypt

**Keywords:** Norovirus, gastroenteritis, RdRp, viral replication, natural compounds

## Abstract

Norovirus (HNoV) is a leading cause of gastroenteritis globally, and there are currently no treatment options or vaccines available to combat it. RNA-dependent RNA polymerase (RdRp), one of the viral proteins that direct viral replication, is a feasible target for therapeutic development. Despite the discovery of a small number of HNoV RdRp inhibitors, the majority of them have been found to possess a little effect on viral replication, owing to low cell penetrability and drug-likeness. Therefore, antiviral agents that target RdRp are in high demand. For this purpose, we used in silico screening of a library of 473 natural compounds targeting the RdRp active site. The top two compounds, ZINC66112069 and ZINC69481850, were chosen based on their binding energy (BE), physicochemical and drug-likeness properties, and molecular interactions. ZINC66112069 and ZINC69481850 interacted with key residues of RdRp with BEs of −9.7, and −9.4 kcal/mol, respectively, while the positive control had a BE of −9.0 kcal/mol with RdRp. In addition, hits interacted with key residues of RdRp and shared several residues with the PPNDS, the positive control. Furthermore, the docked complexes showed good stability during the molecular dynamic simulation of 100 ns. ZINC66112069 and ZINC69481850 could be proven as potential inhibitors of the HNoV RdRp in future antiviral medication development investigations.

## 1. Introduction

Human norovirus (HNoV) is the foremost cause of acute gastroenteritis, affecting approximately 685 million individuals worldwide, including ~200 million children under five years of age, with an estimated 0.2 million fatalities and a social cost of $60 billion each year [1]. While HNoV infection causes a self-limiting sickness in healthy people, it can be lethal in immunocompromised persons, children, and the elderly [2,3]. HNoV infection is the leading cause of mortality in people suffering from viral gastroenteritis because HNoVs are sporadic and highly infectious, and humans are the sole confirmed host. There are presently no treatment alternatives or vaccinations available [4,5], prompting extensive studies into the discovery of antiviral agents that may be utilized for viral infection control and outbreak prevention.

The human HNoV genome is 7.7 kb in size and is organized into three open reading frames (ORF). ORF1 translates polyproteins that are cleaved into non-structural proteins, such as the VPg-like protein, RNA-dependent RNA polymerase (RdRp), and viral proteases [6]. Among them, the RdRp is an important and potential therapeutic target for anti-HNoV drug discovery because of its critical function in viral replication [7,8]. Although only a few HNoV RdRp inhibitors have been discovered, most of these agents have been shown to have little effect on viral replication in cellular systems, presumably due to low cell penetrability and drug-like qualities [9,10]. Hence, the development of anti-HNoV small-molecule therapies or prophylactics is an imperative medical need.

**Figure 1:**
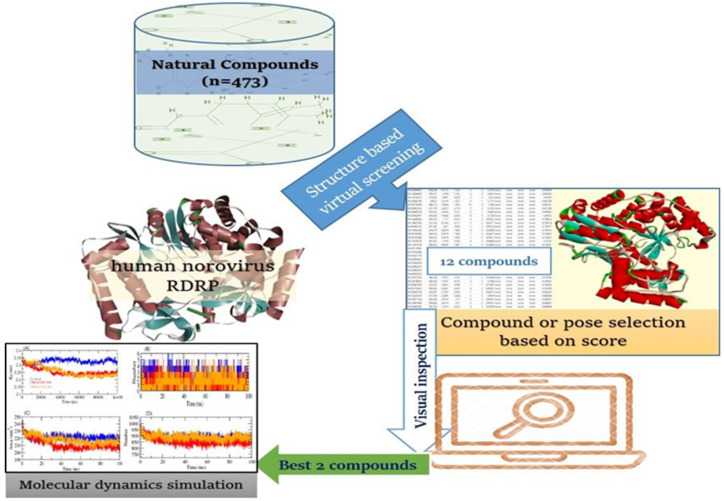
Flow diagram of study design.

The employment of a rapid and lucrative process in the development of novel medication leads has forced the pharmaceutical sector to reconsider its research and development strategy. A computer-assisted drug design technique that makes extensive use of computing power has emerged as one of the most efficient methods of searching for novel lead molecules [11,12,13]. Various computational tools have been developed and used during the last two decades to assist researchers in saving time and cost. These methods involve discovering lead compounds by virtual screening (VS) and computational simulations, as well as chemical and biological data on ligands and molecular targets of possible leads [14,15,16]. The combination of these techniques makes it simpler to reject compounds with attributes outside of ideal ranges and identify viable compounds for optimization [17,18,19,20]. Here, we used high-throughput VS and molecular dynamics studies to identify new natural leads that target the RdRp active sites and could be employed to combat HNoV. The flow diagram representation of this study is illustrated in Figure 1.

## 2. Results and Discussion

RdRp is a multifunctional RNA virus enzyme that is required for viral genome replication and amplification, making it a key target for antiviral drug development [21,22]. Here, we screened a natural compound library against the active site residues of RdRp proteins. Out of 473 compounds, only twelve were found to have better binding efficacy in terms of binding energy than the PPNDS (Table 1). These twelve compounds were further evaluated for their physicochemical properties (Table 2) and visual inspection of their binding poses. We observed that ZINC66112069 and ZINC69481850 are the most appropriate to bind with active site residues of the 4LQ3. All these twelve compounds showed no mutagenic, tumorigenic, reproductively effective, or irritant properties, thereby exhibiting good drug-like potency.

Even the binding energy of a few compounds was found to be higher than these, but in the visual inspection of the binding poses, those were not binding exactly with the targeted residues such as Thr418, Asn505, Ser410, Gln439, and Arg392 [23,24].

**Figure 2:**
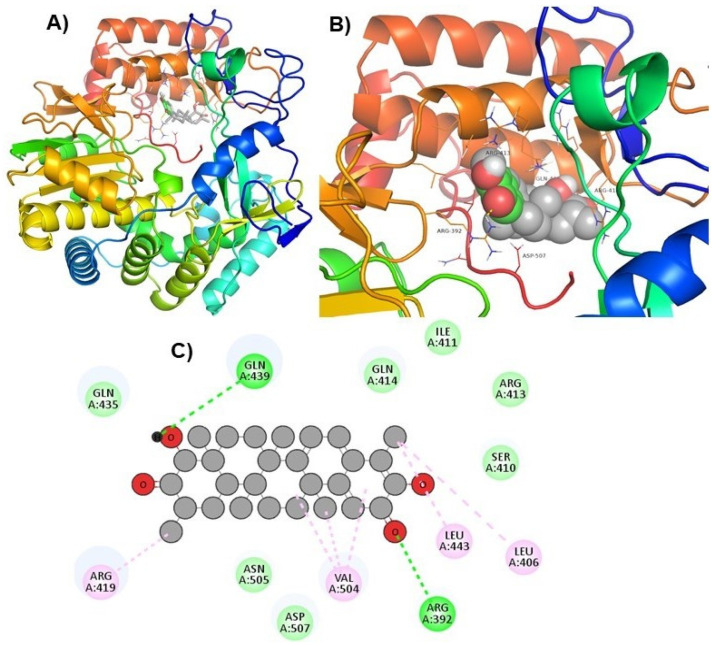
Visualization of ZINC66112069 in RdRp’s active site. 3D views of whole protein and ligand interactions (**A**), 3D views of interacting residues of the RdRp active site with ZINC66112069 (**B**), and 2D views of the RdRp residue interacting with ZINC66112069 (**C**).

**Figure 3:**
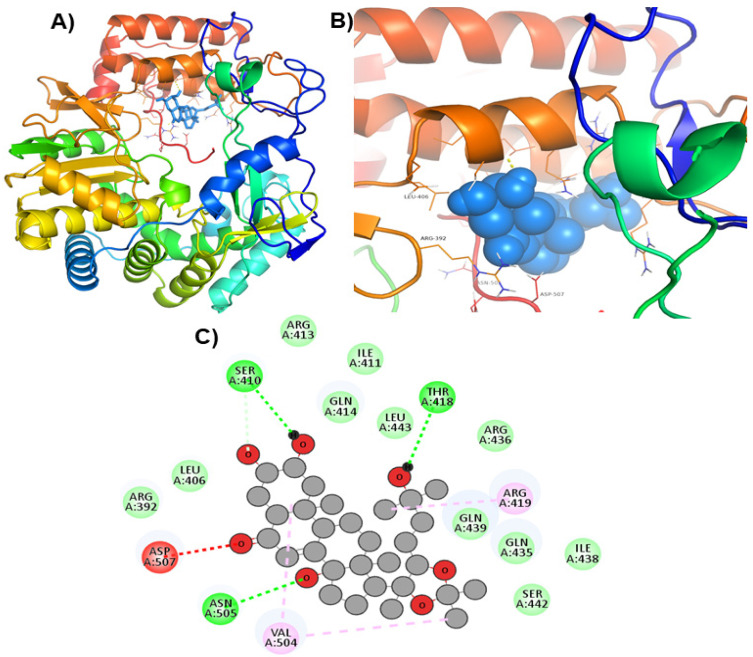
Visualization of ZINC69481850 in RdRp’s active site. 3D views of whole protein and ligand interactions (**A**), 3D views of interacting residues of the RdRp active site with ZINC69481850 (**B**), and 2D views of the RdRp residue interacting with ZINC69481850 (**C**).

Hits ZINC66112069 and ZINC69481850 interacted with key residues of the RdRp protein with BE values of −9.7, and −9.4 kcal/mol, respectively (Table 3). ZINC66112069 interacted with Arg392, Leu406, Ser410, Ile411, Arg413, Gln414, Arg419, Gln435, Gln439, Leu443, Val504, Asn505, and Asp507 residues of RdRp. Of these, Arg392 and Gln439 residues are H-bonded to ZINC66112069, whereas Ser410, Ile411, Arg413, Gln414, Gln435, Asp507, and Asn505 residues interacted with ZINC66112069 via van der Waals interaction (Figure 2). Further, ZINC69481850 interacted with Arg392, Leu406, Ser410, Ile411, Arg413, Gln414, Thr418, Arg419, Gln435, Arg436, Ile438, Gln439, Ser442, Leu443, Val504, Asn505, and Asp507 residues of RdRp. Thr418, Ser410 and Asn505 residues are H-bonded to ZINC69481850, whereas Arg392, Leu406, Ile411, Arg413, Gln414, Gln435, Arg436, Ile438, Gln439, Ser442, and Leu443 residues interact with ZINC69481850 via van der Waals interaction (Figure 3).

**Figure 4:**
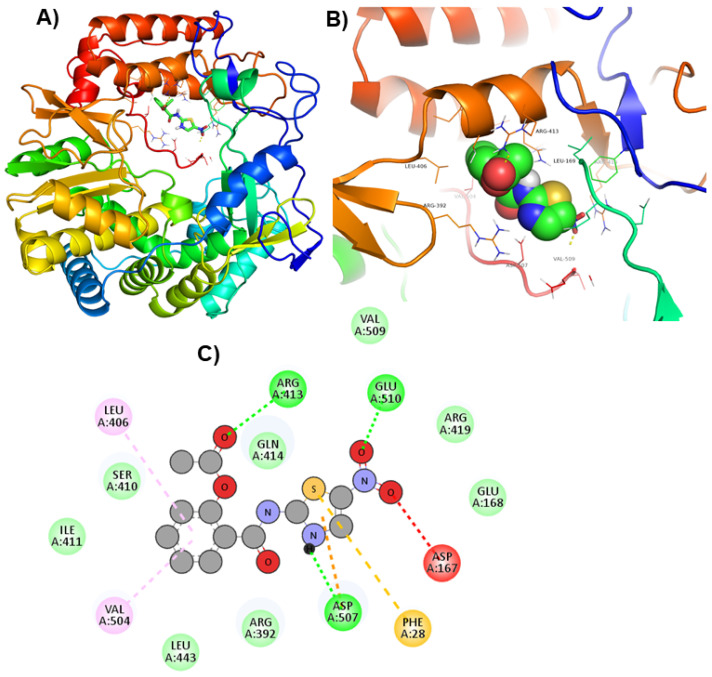
Visualization of PPNDS in RdRp’s active site. 3D views of whole protein and ligand interactions (**A**), 3D views of interacting residues of the RdRp active site with PPNDS (**B**), and 2D views of the RdRp residue interacting with PPNDS (**C**).

Moreover, to get a clearer view of RdRp interacting residues with ZINC66112069, and ZINC69481850, RdRp interacting residues with its co-crystallized molecule (PPNDS) was analyzed by re-docking PPNDS with RdRp. This showed that Asp167, Glu168, Arg419, Glu510, Val509, Arg413, Gln414, Leu406, Ser410, Ile411, Val504, Leu443, Arg392, Asp507, and Phe28 were important in binding with PPNDS (Figure 4). Interestingly, Leu406, Leu443, Ile411, Gln414, Arg413, Ser410, Val504, Arg392, Asp507, and Arg419 were the common residues of RdRp that interacted with ZINC66112069 and ZINC69481850 as well as the PPNDS (Figure 2, Figure 3 and Figure 4), demonstrating that these hits bound at the same pocket of the sRdRp protein as PPNDS.

BE represents the degree of interaction between the compound-protein complex. A high (negative) value implies that the compound binds to its target effectively [25]. Interestingly, hits (ZINC66112069 and ZINC69481850) have higher BEs than that of the PPNDS, revealing that these hits have strong interactions with the RdRp protein.

The hunt for anti-HNoV compounds has long been a priority for medicinal chemists. Various techniques have been used to find possible antiviral compounds to battle the virus [26,27,28]. CMX521 is a therapeutic candidate that inhibits HNoV in mice and is the first nucleoside analog to enter phase one clinical trials to cure human HNoV [10]. The nonnucleoside medication nitazoxanide has been shown in clinical studies to be effective against HNoV. Nonetheless, the specific mechanism against HNoV has remained unidentified to the researchers [29,30]. Other nonnucleoside compounds that effectively inhibit human HNoV include suramin, NF203, and PPNDS. However, the development of these compounds was hampered in the past by toxicity issues. Additionally, modifications to the suramin structure reduced the toxicity while sustaining the ability to effectively inhibit both human and murine HNoV RdRp [31]. Therefore, in the absence of vaccines and conventional medicines, it is imperative to find innovative antivirals that are effective and affordable for the management of viral infections. Natural compounds have been a key source of pharmaceuticals since ancient times, and ~50% of today’s pharmaceutical medications are derived from natural sources [32]. Many natural compounds and herbal substances have been shown to possess potent antiviral action, and their discovery can aid in the development of derivatives and therapeutic leads. These substances have antiviral mechanisms that target interactions between viruses and their hosts as well as viral life cycle stages including replication and assembly [33]. The selected hits in this study are natural compounds that have been suggested to inhibit viral replication by binding with the RdRp of HNoV.

**Figure 5:**
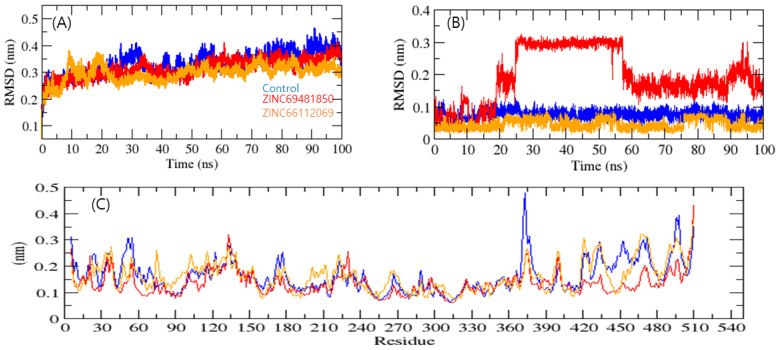
Complex structural stability studies. Protein backbone RMSD (**A**), Ligand RMSD plot (**B**), and RMSF of proteins (**C**). Control, ZINC69481850, and ZINC66112069 were shown in blue, red, and orange colors, respectively.

To determine the stability of a complex, MD simulation studies were performed. The root means square deviation (RMSD) measures protein structural similarity and stability; lower values indicate more stability. RdRp-PPNDS, RdRp-ZINC69481850, and RdRp-ZINC66112069 had RMSD average values of 0.38, 0.34, and 0.25 nm, respectively. The RMSD plot exposed that the RdRp-ZINC69481850 and RdRp-ZINC66112069 complex showed more binding stability than the PPNDS (Figure 5A). The bound structure of the RdRp-PPNDS complex was showing high deviation from its initial conformation, which indicated that the catalytic pocket of RdRp made a quite stable interaction with the screened compound. Further, the ligand RMSD showed that RdRp-PPNDS and RdRp-ZINC66112069 had the least deviation, and interestingly, the RdRp-ZINC69481850 complex showed a high deviation (Figure 5B).

The average fluctuation of all residues, along with the root mean square fluctuation (RMSF) of RdRp during binding with PPNDS, ZINC69481850, and ZINC66112069, were plotted as a function of RdRp residue numbers. The RdRp-ZINC69481850 and RdRp-ZINC66112069 backbones showed steady fluctuations, presumably due to divergent orientations, and RdRp- PPNDS showed high fluctuation found in region 370–380 residues (Figure 5C). On the other hand, RdRp-ZINC69481850, and RdRp-ZINC66112069 complexes showed the overall least fluctuations. It showed that both leads were more stable than the control.

**Figure 6:**
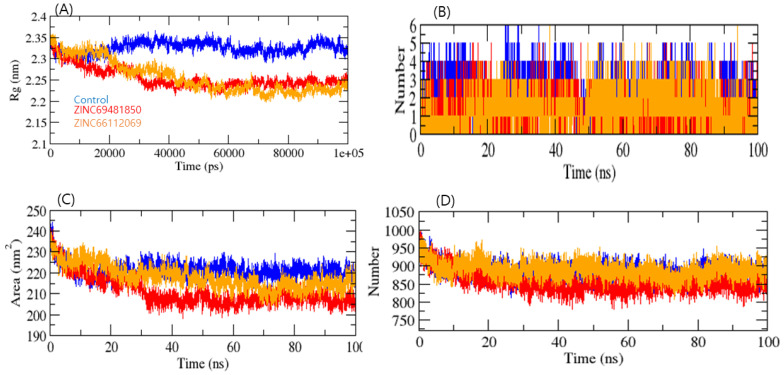
Radius of gyration of complexes (**A**), Number of H-Bonds in complexes (**B**), SASA plot (**C**), and Number of H-Bonds between RdRp and water molecules (**D**). PPNDS, ZINC69481850, and ZINC66112069 were shown in blue, red, and orange colors, respectively.

The Radius of gyration (Rg) analysis was used to gain insight into a biological system’s complex compactness profile. The RdRp-PPNDS, RdRp-ZINC69481850, and RdRp-ZINC66112069 complexes resulted in average Rg values of 2.34, 2.25, and 2.23 nm, respectively. The Rg plot showed less compactness in the RdRp-PPNDS complex than in the RdRp-ZINC69481850 and RdRp-ZINC66112069 complexes. It was inferred that the binding of both compounds made RdRp stable, as RdRp showed fewer Rg values than the positive control (Figure 6A).

Solvent-accessible surface area (SASA) is the part of the surface of a protein that can interact with molecules of its solvent [34]. The average SASA values for the RdRp-PPNDS, RdRp-ZINC69481850, and RdRp-ZINC66112069 complexes were plotted, and the values for the 100 ns simulation for the RdRp-PPNDS, RdRp-ZINC69481850, and RdRp-ZINC66112069 complexes were 228.09, 205.10, and 215.01 nm^2^, respectively (Figure 6C). This analysis indicated that upon binding of ZINC69481850 and ZINC66112069, surface exposure has been reduced, and control increases the surface area of solvent accessibility. Further, H-bond analysis was performed on the ligand-target complex. The binding stability of protein-ligand complexes was established using 100 ns simulations of RdRp- PPNDS, RdRp-ZINC69481850, and RdRp-ZINC66112069 in a solvent environment. The PPNDS and ZINC66112069 showed an average 3–6 H-bond with RdRp protein, whereas the ZINC69481850 showed a 2–4 H-bond (Figure 6B). The complex RdRp-ZINC69481850 and RdRp-ZINC66112069 showed less H-bond interaction with solvent, whereas RdRp-PPNDS showed higher H-bond interaction. It was inferred that the ZINC66112069 might work as a potential drug against the RdRp protein (Figure 6D).

## 3. Material and Methods

### 3.1. Target Protein and Compound Library Preparation

The protein data bank (PDB) was utilized to retrieve the 3D structure of RdRp (PDB ID: 4LQ3). It was then prepared for further screening purposes by assigning bond ordering in ‘Protein Preparation Wizard’ and performing a restrained energy minimization using Discovery Studio (DS) 2021. A library of natural compounds was retrieved from the ZINC database in .sdf format and was prepared using DS and converted to .pdbqt format utilizing the Open Babel tool.

### 3.2. Structure-Based Virtual Screening (SBVS)

VS is useful for expanding databases with active compounds and filtering inactive compounds before they are validated in the wet lab. When combined with other drug-discovery methods, SBVS can lead to interesting results and reduce process costs and time [17]. It uses computational approaches to analyze vast datasets of known 3D structures [35]. The PyRx 0.8 tool [36] was used to screen the prepared library of compounds against the RdRp active site. The protein’s grid center was set to X = −19.528, Y = −25.848, and Z = −2.556. Following the SBVS, extensive interaction analysis and visualization inspections have been carried out, considering the lower binding energy (BE) values, to determine the most stable complex.

### 3.3. Estimation of Physicochemical, and ADMET Properties

The physicochemical properties of the top-screened compounds, as well as their toxicity properties, were predicted using the Osiris DataWarrior software V5.5.0 [37].

### 3.4. Molecular Dynamics (MD) Simulation

Gromacs package 5 of the GROMOS96 43a1 force field [38,39] was used to carry out MD simulations, and the PRODRG server [40] was used to create the topology file for the ligands. MD was performed at 100 ns for each hit-protein complex, and the produced MD simulation trajectories were used for further study.

## 4. Conclusions

In this study, natural compounds were screened against HNoV RdRp using high throughput VS, followed by ADMET prediction and MD simulation analysis. Hits ZINC66112069 and ZINC69481850 bind tightly to the RdRp protein, interact with key RdRp residues, were stable at 100 ns in MD simulation analysis, and have good druglike properties. Further experimental studies are needed to optimize them as RdRp inhibitors.

## Figures and Tables

**Table 1:** List of compounds with higher binding affinity than the positive control.

Ligand	Binding Energy (Kcal/mol)
ZINC69482526	−9.9
ZINC69482529	−9.9
ZINC66112069	−9.7
ZINC69481856	−9.6
ZINC69482059	−9.5
ZINC69481850	−9.4
ZINC69482023	−9.3
ZINC69481956	−9.3
ZINC69482024	−9.3
ZINC69482510	−9.2
ZINC04097720	−9.1
ZINC69482364	−9.1
PPNDS	−9

**Table 2:** Physicochemical and drug-likeness properties of selected compounds.

Molecule Name	Mol Weight	cLogP	cLogS	H-Bond		Drug Likeness Score	Mutagenic	Tumorigenic	Reproductive Effective	Irritant	Drug Score
				Acceptor	Donor						
ZINC69482510	392.493	4.5121	−4.402	4	1	1.0176	N	N	N	N	0.51537
ZINC66112069	436.59	4.4475	−4.742	4	2	0.4587	N	N	N	N	0.433376
ZINC69482023	414.651	4.4768	−5.443	3	2	0.37333	N	N	N	N	0.312927
ZINC69482526	544.639	1.2624	−4.575	9	2	−2.3645	N	N	N	N	0.294769
ZINC69482059	497.737	4.4729	−6.538	4	0	−0.333	N	N	N	N	0.255883
ZINC69482024	430.65	3.5501	−4.936	4	3	−2.6117	N	N	N	N	0.248718
ZINC69481856	532.631	3.1858	−5.326	7	0	−3.4332	N	N	N	N	0.238752
ZINC69482529	613.769	2.2649	−5.844	9	2	−1.6313	N	N	N	N	0.220738
ZINC69481956	472.707	5.4045	−5.643	4	2	0.36826	N	N	N	N	0.177611
ZINC69481850	520.704	3.2548	−4.713	7	4	−0.02161	N	N	N	N	0.14069
ZINC04097720	426.726	7.5888	−6.968	1	0	−3.3053	N	N	N	N	0.132645
ZINC69482364	424.754	8.5403	−7.379	0	0	−7.4295	N	N	N	N	0.119384

**Table 3:** 2D structure of hits and list of H-bonded residues of RdRp-hits complexes.

Compound	Structure	H-Bonded Residues	Van der Waals Interactions
ZINC66112069	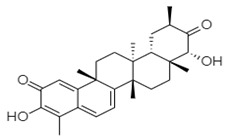	Arg392 and Gln439	Ser410, Ile411, Arg413, Gln414, Gln435, Asp507, and Asn505
ZINC69481850	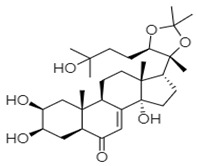	Ser410, Thr418, and Asn505	Arg392, Leu406, Ile411, Arg413, Gln414, Gln435, Arg436, Ile438, Gln439, Ser442, and Leu443
PPNDS *	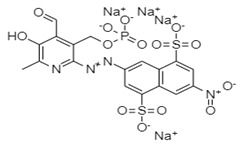	Arg413, Asp507, and Glu510	Glu168, Arg392, Ser410, Ile411, Gln414, Arg419, and Val509,

* Positive control.

## Data Availability

The data presented in this study are available in this article.

## Abbreviations

HNoV: Norovirus, RdRp: RNA-dependent RNA polymerase, VS: Virtual screening, DS: Discovery Studio, SBVS: Structure-based virtual screening, BE: Binding energy, MD: Molecular dynamics, RMSD: Root mean square deviation, Rg: Radius of gyration, RMSF: Root-mean-square fluctuation, SASA: Solvent accessible surface area.
